# A formative study of disposal and re-use of old mosquito nets by communities in Malindi, Kenya

**DOI:** 10.5281/zenodo.10870353

**Published:** 2015-06-29

**Authors:** Lydiah W. Kibe, Anne W. Kamau, John K. Gachigi, Annette Habluetzel, Charles M. Mbogo

**Affiliations:** 1 KEMRI Wellcome Trust Research Programme, Kilifi, Kenya; 2 University of Nairobi, Institute for Development Studies, Nairobi, Kenya; 3 Ministry of Labour, Social Security and Services, Nairobi, Kenya; 4 University of Camerino, Camerino, Italy

## Abstract

**Background:**

About 30 million insecticide treated mosquito nets have been distributed in Kenya since 2001 and ownership is approaching full coverage. As a consequence of this achievement, Kenya is faced with the challenge of disposing old mosquito nets that are no longer in use. The study aimed at investigating ways of disposal and re-use of old and torn nets by end users.

**Materials and Methods:**

A formative study was conducted in the former Malindi District, which is comprised of Malindi and Magarini sub-counties of Kilifi County in Coastal Kenya. A total of 6 Focus Group Discussions, 10 Key Informant Interviews and 9 transect walks/drives were undertaken. Data from the different sources were analysed separately and triangulated for similarities and differences.

**Results:**

There were variations in disposal and re-use of old nets between urban and rural or peri-urban residents. In all settings, people adopted innovative and beneficial ways of re-using old, expired nets, and those that were damaged beyond repair. Common causes of damage were fire, children, domestic animals sharing the sleeping room and friction from the bed poles while hanging or tacking it in under a sleeping mat. Re-use was most prominent in farming activities (78%) and less to for use in mosquito control, like window screening (15%). The remaining 8% was related to making ropes, swings, footballs, goal posts and fishing nets. Advantageous texture and nature of the netting material, perceived economic benefit and lack of guidelines for disposal were the main reasons cited by residents for re-using old nets.

**Conclusions:**

It is important that re-use and disposal of old mosquito nets is distinguished from misuse of newly distributed mosquito nets. Alternative uses of old nets as opposed to misuse of new nets was found to be common in our study.

## 1 Introduction

In the past decade, there has been a remarkable increase in international funding for malaria control. This led to tremendous progress in population-wide access to insecticide -treated mosquito nets (ITNs) in Kenya and globally. Originally, in the 1990s, nets had to be treated with insecticides periodically by their owners but following the development of Long Lasting Insecticidal Nets (LLINs) in 2007 this operational disadvantage was overcome and LLINs became recommended by the World Health Organization for universal coverage, i.e. LLINs should be used by all persons at risk [1,2]. According to WHO world malaria report of 2010 [3], by the end of that year approximately 289 million LLINs were distributed across sub-Saharan Africa (SSA), about enough to cover 76% of the 765 million persons at risk of malaria. By 2012, 117 countries, including 34 in Africa, had adopted the WHO recommendation to provide LLINs for free to all persons at risk of malaria. From the world malaria report published in 2013 [4], it emerged that 88 countries, including 39 in Africa, distributed LLINs in 2012 free of charge. This led to a remarkable increase in net coverage, with about 54% of households in SSA owning at least one LLIN by 2013 [4,5].

LLINs are estimated to have a useful life of up to four years [6-8] that may be even longer due to the insecticidal residual efficacy [9]. However, physical deterioration of the net may shorten the effective lifespan to 1.5-2.5 years [6,10-12]. When nets are physically deteriorated and insecticidal residual activity is reduced by improper washing practices, they offer poor protection to the users [13,14]. Thus, proper net care and repair practices must be promoted [15].

In Kenya, over 30 million insecticide-treated mosquito nets have been distributed to vulnerable populations since 2003 and more recently to all persons at risk of malaria [16]. Since 2002 a number of strategies have been adopted to enhance net distribution. These are distribution of highly subsidised nets through social marketing by urban and rural retailers in malaria-prone districts and distribution of heavily subsidised nets to pregnant women and children under five years of age through maternal and child health clinics. Non-subsidised nets are sold to the public through the commercial retail sector. Other short term distributions include those organised by specific projects and by non-governmental organisations (NGOs). A major contributing factor for achieving high levels of coverage were mass distribution campaigns. In Kenya, such campaigns were conducted in 2006 and 2012. In 2006, the country saw the distribution of 3.4 LLINs free of charge to children below five years of age in malaria prone districts. In the 2012 campaign, 11.5 million LLINs were distributed free of charge to all inhabitants living in the 87 malaria-prone districts in the country. This distribution aimed to achieve universal coverage whereby one net was given to every two household members. In cases where households had an uneven number of people, the number of nets issued was rounded upwards [17].

These diversified net distribution strategies resulted in a rapid increase in net ownership, from 5.9% in 2003 to 50.2% in 2006 [4,18] to 83.4% in 2013 [19]. Malindi, like other parts of Coastal Kenya, received two lots of free mosquito nets, the first in 2006 and the second during the 2012 campaign. With such massive distributions of LLINs but without guidelines for disposing old and torn nets at the household level, it remains a question as to what households do with such nets that are no longer used when they receive new ones, especially after mass distribution campaigns as was the case in Malindi in 2012.

While there are a number of reports on mosquito net re-use and misuses, most of these lack scientific data to support their claims [20]. There are, however, some studies that have reported the use of intact and effective nets as sleeping mats, as fishing nets or for drying fish in Kenya and Zambia [21-23], or as bridal veils in Uganda and Tanzania [24,25]. In addition, people use their creative thinking to come up with practical, alternative uses of old or torn nets. Here we describe the ways that communities of Malindi sub-County in Coastal Kenya use old and worn nets to improve their livelihoods as opposed to viewing these nets as trash.

## 2 Materials and Methods

### 2.1 Study area and population

The study was carried out one year after the 2012 mass distribution, from August to December 2013 in Malindi sub-County on the coast of Kenya. Previous studies have described the area in detail [26-29]. Briefly, Malindi town is located 120 kilometres North of Mombasa, Kenya’s second largest city. The main economic activities include farming, fishing, tourism and small-scale business. Malindi has a population of about 400,000 people [30]. The study utilised the sampling frame developed by Keating and colleagues in 2003 [26] to select study areas. According to their framework three types of areas were selected, namely urban, peri-urban and rural areas [26]. The urban area was characterised by a high population density, plenty of commercial activities and residential houses with little surrounding vegetation. This area was also relatively well supplied with electricity, paved roads, and piped water. The peri-urban area was located on the outskirts of the urban area and was characterised by small-scale farming and few residential houses. Little or no drainage system was present. Finally, the rural area was distinctively characterised by a low population density and the majority of the population living in clustered villages with mud and thatch houses and large land areas utilised for agricultural purposes. Having received LLINs from the universal coverage campaign in September 2012 was a further inclusion criterion for the selection of one study village per area. With the help of local leaders and public health staff, three villages namely Maweni, Maisha Mapya and Kavunyararo were selected to represent the urban, peri-urban and rural settings, respectively. Maweni is located about 2 km from the shores of the Indian Ocean. Most residents are engaged in informal businesses and low paid jobs. Urban farming is also common. Maisha Mapya is located about 6 km from the Indian Ocean. There are a few residential houses with peri-urban farming carried out in a much larger area than in Maweni. Finally, Kavunyaralo is about 25 km from the Indian Ocean. The majority of the residents are small-scale subsistence farmers and a few engage in trading.

As shown in Table 1, a qualitative approach was deemed most appropriate due to the fact that there is very little existing research that has been conducted this far on this topic. The methods used included focus group discussions (FGDs), key individual interviews (KII) and transect walks/drive. FGDs were conducted in each selected village in the study area. Participants were purposely selected with the help of criteria developed by the researcher. The criteria were set to include men and women aged between 18-70 years of sound mind who a) had an old mosquito net being used for alternative purposes in their compound or farm, b) whose compound/ farm had no alternative net use practices. In each of the 3 areas, two FGDs were conducted, and each FGD comprised 6 - 11 participants. The discussions were conducted by 2 experienced social scientists (male and female), a note taker and an observer. A focus group (FG) guide (described below) was used to moderate the discussion. One FGD was conducted per day to allow for transcription and reflection of the information identified in the transcripts. This gave the researchers the opportunity to refine the session guide and prepare for subsequent interviews. In total, the 6 FDGs involved 20 men and 31 women. The interviews were conducted at a school, a community hall or under a tree.

**Table 1.** Number of respondents and observations per area and by method

Study area	FGD			KII			Transect Walk/Drive
	Male groups	Female groups	Total	Male	Female	Total	
Urban	1	1	2	2	2	4	3
Peri-urban	1	1	2	1	2	3	3
Rural	1	1	2	1	2	3	3
Total	3	3	6	4	6	10	9

Key Informant Interview (KII) respondents were purposely selected using a snowballing method. The interviewees included public health officials, opinion leaders such as village elders and chiefs, community health workers and community volunteers. A session guide (described below) was used for the interviews lasting about 1– 1.5 hours. A total of ten KIIs were conducted by an experienced social scientist assisted by a note taker. Net disposal or re-use practices were also assessed by direct observation.

FG and KII guides were used in data collection. Developed guides were pre-tested and according to the outcomes, questions were re-defined. The guides were translated into Giriama and Swahili and back translated into English. They focused around questions concerning “old mosquito nets”, how old is an old net and what makes a net to become due for disposal, who determines on disposal or re-use, how and when. Where to take the old, expired and torn nets; what motivates residents to re-use mosquito nets? What are people’s suggestions for improving net disposal practices? The discussion guides were used to moderate sessions in FGDs and KII. Data collection progressed from transect walks to FGD to KII. This enabled us to identify emerging themes and issues that required further probing.

Transect walks/drives were undertaken by the research team in each of the study areas. A pre-tested tally sheet was used to collect data on uses of nets and type of nets observed during transect walks/drives. Before embarking on a transect walk/drive, the research team studied the maps of study villages and roads, foot paths and land marks. This exercise was facilitated by team members who lived or previously worked in these villages. The team agreed on the foot paths and roads which represented a cross sectional line through the village and those that had the likelihood of finding numerous net re-use practices. The team followed the footpaths/roads recording all mosquito net re-use practices observed and tallied according to the category of re-use and type of net used. The guide allowed for inclusion of new re-uses observed and later, these re-uses were assigned to a category. The team also took pictures to document the various practices observed. In total, nine transect walks were performed, 3 in each study site which included two transect walks and one transect drive per site.

### 2.2 Training of research team

Three (3) field assistants were recruited on the basis of their previous experience of working in the community or being a resident of Malindi. All had (at least) completed secondary education. They underwent three days of training, which was organised as follows: one day theory on the research tools used in the study and their correct application, communication skills, consenting process and recording responses; one day was dedicated to practical simulation exercises on data collection tools used in the study and one day to pretesting, reviewing and adjusting of the discussion guides and transect walk tool.

### 2.3 Data management and analysis

All audio tape recorded materials of FGDs and KII were transcribed and observers’ and summary notes of all discussions were compiled daily. All transect walk tally sheets were entered in excel sheets. Pictures taken during transect walks/drives were stored in a picture folder together with other pictures taken in the field. The two social scientists who were experienced in qualitative data analysis independently coded the transcripts and notes using a hybrid approach of inductive and deductive coding. The two coders later agreed on the codes and categories and the underlying meaning of the different categories was formulated into a theme. Atlas.ti version 6.2 software was used to organise the data.

### 2.4 Ethical clearance

Ethical clearance for the study was obtained from the Ethical Review Committee of the Kenya Medical Research Institute and was assigned KEMRI ERC No: 2277. All respondents were provided with information regarding the purpose of the interviews and verbal consent was sought before data collection. The reason for tape recording the discussions was explained and consent was sought before commencing of the interviews.

## 3 Results

### 3.1 Demographic characteristics of participants in FGDs

The size of the focus group discussions ranged from six to eleven participants, with an age range of 18 to 70. Most of them were married and engaged in small scale businesses and farming. Various basic socio-economic and demographic indicators were collected prior to the start of the discussion groups (Table 2).

**Table 2.** Socio-demographic profile of participants in focus group discussions

Category	Sub-Category	Number (n=51)	Percentage (%)
Sex	Male	20	39
	Female	31	61
Age	19-35	18	35
	36-70	33	65
Marital status	Married	41	80
	Single	8	16
	Others	2	4
Educational status	No education	6	11
	Primary	32	63
	Secondary	12	24
	College	1	2
Main Occupation	Farmers	25	49
	Employed	14	27
	Unemployed	12	24

### 3.2 Primary themes generated from the data

Six themes emerged from analysis of the data sets: (1) sources of the nets (2) factors determining disposal of nets (3) motivations and benefits to re-using old nets (4) disposal mechanisms and re-use of old nets (5) misuse verses alternative uses (6) ways of improving disposal mechanisms.

#### 3.2.1 Sources of nets

The sources of nets mentioned included: mass distribution campaigns (2006 and 2012), child welfare clinics [free nets to pregnant mothers and children under one year during the first visit], and shops.

*We got nets from the campaign (2012). They were many like Kenya uniform. Whenever you go, you saw people carrying them….(Urban FGD)*

*At the clinic, only pregnant woman or small children of less than one year are given mosquito nets. An old man like me have to buy one from the shop. And you know at the shop it is expensive. ….it ranges between Ksh400- 800 (€ 4-8) depending on the size and shape of the net (Rural FGD)*

#### 3.2.2 Factors determining disposal of a net

Participants in FGDs were asked how they decided a net is ready for disposal. From their discussion, it was evident that the physical condition of a net and not necessarily the age of it was the main consideration. Most of the respondents indicated that the age of the net did not matter, as long as it was still in a good condition and the user did not have another net to replace it. In most cases, respondents described the condition of the net in reference to the number and size of holes, and repairs made on the nets. Most damages were cited to originate from burning by tin lamps, friction from the mat and edges of the bed, sparks of fire, sticks from the house, children playing with the net and friction on the edges of the bed and goats urinating on the nets thus necessitating frequent washing.

*My net had big holes like my fist and mosquitoes were entering through these holes. I had to buy another net to replace it (Urban FGD)*

*If the holes are many and big on the net, you do not sleep since mosquitoes come looking for you through the holes as if you are sleeping without a net. So you are forced to either buy another one or sleep without a net.* (*Peri-urban FGD*)

*Sometimes you repair the holes until the net cannot be repaired anymore. You repair it today, after a week you find another bigger one. If it is old you throw it away in the trash and get a new one or stay without one (Urban FGD)*

*Our houses are like you can see them (referring to mud thatched houses). We sleep with our chicken and goats inside the house. This makes the nets get dirty very fast as the goats sometimes urinate on the nets. You have to wash it regularly and this makes it get torn very fast (Rural FGD)*

#### 3.2.3 New net versus old net

The majority of the respondents said their nets had expired and needed to be replaced. They indicated that their nets were already old enough to be disposed of and the availability of the new nets following the distribution exercise gave them the opportunity to replace them. Those whose nets were still intact and in good condition said they continued using them while some kept them in the house for visitors and for future use in case the new one got damaged.

*You see the government takes time before they bring us new nets. The last time we were given nets was 2006. Since that time until the other day (2012) we were using the old nets. I am grateful I got a net to replace my old one (Respondent peri-urban)*

*As you walk in this village, you will see old nets everywhere….in the trash areas, on the roof of houses, fences, urinal and many other places. The owners got new ones and replaced them and they did not know what to do with the old ones. (Urban FGD)*

Another respondent said *old net is trash, what can one do with a torn old net? (Urban FGD)*

#### 3.2.4 Disposal mechanisms and re-use of old and torn nets

**Figure 1.**
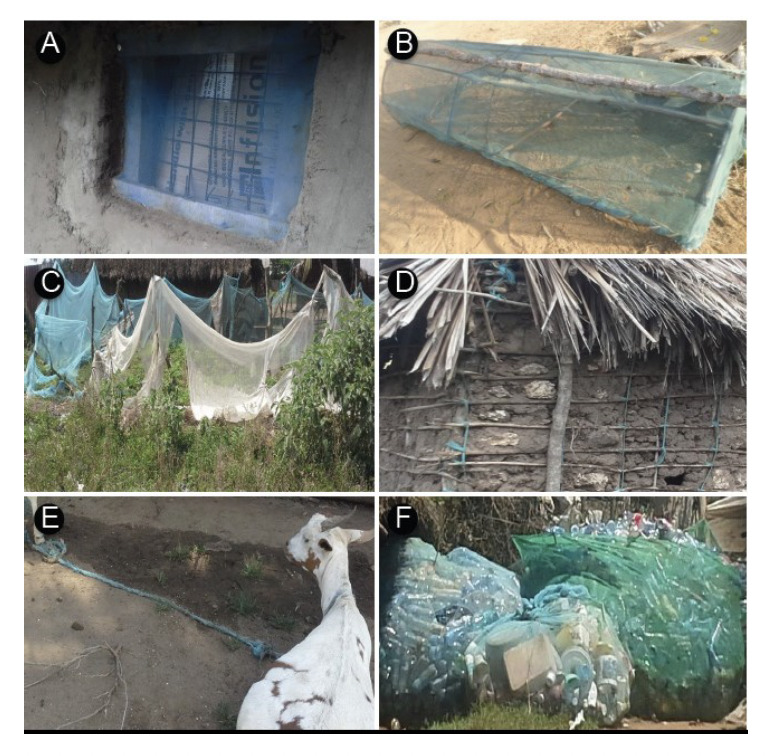
Uses of old and worn bednets (LLINs) by Malindi residents. A: Use for window screening, B: Chicken pen, C: Securing kitchen garden, D: Use in house construction, E: Rope for tethering animals, and F: As storage bags for plastic bottles.

As shown in Table 3, a total of 424 observations were made on the alternative uses of old mosquito nets during the transect walks/drives. Re-use of old nets was more frequently observed in rural areas (46%) and peri-urban areas (37%) than in urban areas (17%). Most of the re-uses were related to farming practices and security needs, namely old nets were employed for reinforcing fences and shelters (25%), net ropes for tying animals, building and furniture materials (23%), protecting seedlings (17%) and chicken coops (13%) (Figure 1). Importantly, the residents used the old nets for mosquito control in screening windows (11%), or for covering wells and water containers (4%). Other alternative uses included applications in leisure activities with children using the nets to make goal posts, playing balls, strings and jumping ropes and swings. Women used the old nets to make *hando*, a traditional attire for enhancing their buttocks to look more attractive. The attire is made using stripes from old clothes or sisal materials. More uses were reported during the FDGs such as material for scrubbing utensils and body care like a sponge, or ropes for making a traditional house (Table 4). Polyester net material in particular was perceived to be tough and long lasting. When constructing a traditional (coastal) house, locally available materials such as wood to support the structure, tree branches for the walls, palm leaves or grass for the roof, sisal strings for tying the wood and the palm leaves are needed. With the availability of old nets, polyester netting cut into small stripes was used as ropes for tying together the branches and the wood to make the main structure of the house and the wood with palm wine leaves for the roof. This helped to avoid the cost for buying sisal rope which is expensive and sometimes out of stock. The stripes of the nets were also used by carpenters for making Miji Kenda beds and Swahili chairs. Most people preferred furniture manufactured from ropes of old netting because the fabric is considered strong and durable.

**Table 3.** Observed alternative uses of old mosquito nets in Malindi, Kenya

Observed uses of old nets	Urban	Peri –urban	Rural	Total	%
Covering/reinforcing (fence, mnazi dens, bathing shelter)	23	37	46	106	25
Ropes (animal, clothes lines, building, Mijikenda beds and chairs)	9	39	50	98	23
Protecting seedlings/plants	7	27	36	70	17
Chicken coop	8	18	30	56	13
Window screening	17	15	14	46	11
Leisure (goal post nets, children swings, balls)	4	4	3	11	3
Covering well/water containers	6	8	3	17	4
Fishing	0	3	5	8	2
Hando	0	2	4	6	1
Other	0	3	3	6	1
Total	74	156	194	424	100

**Table 4.** Re-uses of old bednets in relation to context

Context	Specific uses
Mosquito control	Torn bednets were cut into sizes suitable for window screening to prevent house entry by mosquitoes, or for covering open wells or water storage containers (tanks) preventing mosquito breeding and dirt entering the water.
Farming	This included covering for chicken and ducks coops to protect chicks against predators and restrict adult birds ones from loitering around and destroying crops. Protecting seedlings or plants from getting damaged, or use as ropes for tying animals to restrict them while grazing and tethering in their shed. Used to cover drying maize and cereals to protect from animal damage.
Domestic	Make ropes for the clothes lines, make cleaning material for body scrubbing and dish washing sponges.
House improvement	Cut into pieces that fit into window curtains to offer privacy in the room, ceiling material to prevent falling materials on the bed using the material, and room divider to provide privacy in the room.
Building and furniture	Ropes for construction of mud thatched houses, Miji Kenda beds, or Swahili chairs.
Traditional women’s attire	Traditional attire (hando) worn by women when going to the market and functions such as weddings, public meetings, or funerals. The attire is worn to draw attention and to be admired by men. The cultural practice recognises women with big buttocks.
Recreation	Children skipping ropes, improvised swings by children and goal post net.
Security	The nets were used to fence around the homes, palm wine drinking dens to secure the compounds and restrict entry.

In addition, more than 90% of the nets re-used for alternative purposes were identified as *“the blue rectangular and conical net with big holes”.* This description suited nets that were distributed in 2006. The respondents in FGDs said they used these nets because the netting is strong and durable. As commented by a respondent during an FGD session in the rural area who made a rope to secure her animals during grazing:

*I made ropes for my goats and cows. Those ropes are better than the ones we buy in the market. I have used it now for four months and they are still in a good condition.* (*Rural FGD*)

#### 3.2.5 Motivation to re-use old bednets

A number of reasons emerged from the FGDs regarding the residents’ choices to re-use old bednets for alternative purposes. The main reason was the material fabric of the net which was considered to be strong and durable. Others reasons given were availability of old nets and saving costs of alternative materials. The majority of the participants stated that they did not know what to do with the old nets when they received the new ones during the 2012 campaign. It was also reported that at the time of receiving the new nets, they were not informed what to do with the old ones. The following quotes are examples from the residents’ comments on their motivations to re-use old nets:

*We re-use the old mosquito nets because they are old and most of them are torn beyond repair. We are happy we received the new ones to replace them. Since we do not know any better ways of disposing the nets, we use them for what we think is helpful to us. We have not been told other better ways than what we are using them for. (Rural FGD)*

*When we were given the new nets, we did not know what to do with the old nets. We used our wisdom to make appropriate use of the old nets. If you have any other better ways, we will be happy to listen to you” (Rural FGD*)

According to a key informant, there was lack of a plan or strategy for disposing old nets by the responsible government institutions. He cited the challenges with finances, human resources, storage facilities and even ideas on what to do with the old nets after collecting them. It also emerged that the National Environmental Management Authority (NEMA) is responsible for setting up and enforcing laws and regulations to manage plastic waste including disposed bednets. However comments from the residents indicated that they know little about NEMA.

*We know the Ministry of Health, not NEMA. There are some people from the Ministry of Health who come around if they find a net being employed for other uses like covering chicken or vegetables, they caution you and order you to stop or they report you to the chief. If you are taken to the chief, they say you will be fined for misusing the net (KII 4)*

The lack of guidelines for disposing the nets upfront was the major challenge raised by various respondents. This was confirmed in a KII with a staff member of the Malaria Control Unit (MCU), formerly the Division of Malaria Control:

*We acknowledge the problem of disposal of old nets and this is because we lack the guidelines for old net disposal (KII6)*

*We need to work together with NEMA on these guidelines. We will do our part but it is their mandate to enforce proper disposal of waste (KII 2)*

#### 3.2.6 Users perceived benefits on the various uses of old nets

Generally, residents said old nets offered them an alternative material which was useful and appropriate to a variety of their needs:

*The old nets benefited farmers, carpenters, builders, children, housewives and almost everyone” (Peri-urban FGD)*

The main benefit observed to urban residents, was in reusing old net material to fit it over open wells and also to screen windows. This was done to prevent mosquitoes from entering inside houses and also to prevent mosquito larvae from breeding and adults from resting inside the well. Nets were also used as curtains to divide the room for privacy reasons and as ceiling to prevent falling dirt and debris above the bed.

The rural and peri-urban residents had more benefits in farming activities such as chicken coops, protecting the plant nursery and other plants. They also used them for covering cereals such as maize when drying it to protect against animal damage. Other uses were making ropes which were useful for making clothes lines, building traditional houses, tying animals to restrict their movement while grazing or in the shed, children skipping ropes, making Swahili chairs and traditional Giriama beds. Table 4 summarises various uses of old nets.

In contrast, FGDs and the transect walks showed clearly that households in urban areas discarded old nets (trash) or burned them. Observations from the transect walks saw discarded old nets scattered all over and this formed part of the non-biodegradable household waste polluting the environment. It was reported by urban participants that, following the distribution of the campaign nets, old nets were seen scattered everywhere in the surroundings.

## 4 Discussion

Our results show that factors that determined replacement of a net were mostly related to the physical condition, age and availability of a new net. Physical condition entailed the presence of holes (number and size) in the nets, which permitted mosquito entry. The results also show that availability of a new net was a motivation to replace old and torn ones. Our findings show that the majority of the new and torn nets were replaced after a free mass distribution campaign which aimed at achieving universal coverage. It was also observed that the majority of the nets that were re-used for various purposes were old, expired or torn beyond repair.

In contrast to other studies on the ‘misuse of bednets’ our study revealed the imaginative, creative, and innovative ways of recycling old and torn nets by household owners in Malindi, Kenya. The alternative uses of the old and torn nets employed were driven by residents’ needs and priorities, and necessitated by lack of official guidance on how old nets should be disposed of after expiry. As shown in Tables 3 and 4, the residents of Malindi made use the old nets for purposes that in their perception were beneficial to them; these purposes included window screening, reinforcing fences*,* tying animals*,* making chicken coops*,* making ropes for different uses such as house construction, using the nets as a sack for storing plastic materials after collection before they are taken for recycling (Figure 1). Ropes were also used to make Swahili chairs and Miji Kenda beds. Other uses included recycled material as cleaning materials (brush, rough sponge) for body care and washing dishes. Similar to studies conducted elsewhere, bednets have been reportedly used for a range of purposes not related to mosquito control. Studies conducted in various countries found nets used for a variety of purposes such as protection of cabbage production in smallholdings in Benin [31]; prawn fishing in the Solomon Islands [32]; protection of seedlings and garden crops and plaited into string to tie cattle in Ethiopia [33]; as washing sponges in Liberia [34]; protecting seedlings, covering meat in butcheries, filtering water and as window curtains in Senegal [35]; fishing and protecting crops in Timor Leste [36]; as curtains or for protecting chickens in Tanzania [37]. These alternative uses, put in practice already in a substantial number of countries, may be a starting point for developing alternative disposal guidelines for old and expired nets suitable for different settings.

Only a few studies reporting on alternative uses of nets are providing evidence on net condition at the time of re-purposing [35], so that it is difficult to judge to what extent people misused functional nets or re-used expired ones. Based on our study, bednets employed for alternative uses were torn or expired and were no longer useful for mosquito control. These nets could not be classified as ‘misused nets’ since this would mean use of newly distributed nets or intact nets that would have been useful for mosquito control. The study revealed that the re-used nets were received 6 years before the mass distribution campaign of 2012. These findings support other research findings that it is primarily old nets or nets torn beyond repair that are utilised for alternative purposes [35,38]. Contrary to a study conducted by Loll *et al.* [35] who reported that the availability of a new net was unlikely to be a trigger for discarding an existing net in decent condition by residents of Louga (Senegal), our results showed that acquisition of a new net was the main reason for replacing the old net. This is explained by the availability of lots of old nets after the 2012 mass distribution campaign, when nets were given for free with the aim of achieving universal coverage. However, not all nets replaced with new ones were discarded or re-used. Some respondents who replaced nets that were in good useable condition kept them as spare nets for visitors or as an extra net for replacing the new one when it would get damaged. This suggests that availability of an extra net in the household was perceived to be important and thus often the net was reserved for future use. This behaviour reveals inadequate knowledge on the importance of the insecticidal component of LLINs. It shows that people are not aware of the fact that the insecticide loses its residual activity over time and that nets without the active chemical provide only marginal protection. It is therefore critical for programmes involved in net distribution to diffuse messages that encourage residents to continue using their old nets that are in good useable condition but not for longer than 5 or 6 years. Also, when continuing to use the old nets, people should avoid opening the bag of the new one for the insecticide to maintain its efficacy.

As shown in this study, the majority of the re-use modes provided evidence benefits to the population. Therefore, instead of condemning the residents, health officials should provide guidance on the best practices that households should follow regarding the use of old nets for mosquito control. Such examples can be drawn from this study where the residents re-used the old nets for screening windows, covering water storage containers, wells and as ceiling materials. This should be promoted and encouraged since the nets will not only prevent the residents from nuisance biting but may also contribute to control the proliferation and bites of other mosquito species responsible for the transmission of filariasis, Dengue fever, Chikungunya and Rift Valley fever which have been reported from this area [29,39]. Since some nets have been found to be damaged before their effective lifespan ends [40] but still exhibit residual insecticidal activity, once used as window and door curtains, these can still offer partial protection to residents for the prevention of malaria and other mosquito borne diseases.

Similar to observations made by Eisele *et al.* [20], the use of old mosquito nets in Malindi did not negatively affect malaria control efforts since the majority of the residents had received new nets during the campaign. Therefore, the various strategies used by this community in reusing the old mosquito nets can be a starting point for the development of guidelines for net disposal at household level. On the other hand, for maintaining a high level and proper use of bednets in communities, continuous education to reinforce the importance of the nets for malaria control remains fundamental.

The main reason for disposing the net in this study was it’s physical condition occasioned by a conspicuous number of holes and the presence of large sized holes. Although residents of Malindi are repairing damaged nets by stitching the holes, they seem to have big challenges in keeping their nets intact due to the numerous environmental risk factors. Communication campaigns tailored to local situations with demonstrations and scenarios on how to avoid such risks should be implemented in the communities. This may help in increasing proper net utilisation and reducing the number of nets that are used in alternative ways before they expire.

## 5 Conclusions

The alternative uses of old and worn out nets should not be indistinctly interpreted as misuse. Rather, this should be seen as an innovative way of using expired nets in the absence of proper guidelines for net disposal. It is important to promote viable ways of using old and torn nets, in particular for uses that contribute to mosquito control. However, care must be taken to clarify their complementary value in respect to the LLIN strategy. Possible options could be to find community-led means of collecting old and torn nets and use them to fabric window curtains or covers for water wells. Last but not least, old nets could also become an additional source of income for communities by encouraging collection, sorting and making ropes for the ready market and other uses that were reported in this study.
